# Dense RGB-D Semantic Mapping with Pixel-Voxel Neural Network

**DOI:** 10.3390/s18093099

**Published:** 2018-09-14

**Authors:** Cheng Zhao, Li Sun, Pulak Purkait, Tom Duckett, Rustam Stolkin

**Affiliations:** 1 Extreme Robotics Lab, University of Birmingham, Birmingham B15 2TT, UK; R.Stolkin@bham.ac.uk; 2 Lincoln Centre for Autonomous Systems (L-CAS), University of Lincoln, Lincoln LN6 7TS, UK; lisunsir@gmail.com (L.S.); tduckett@lincoln.ac.uk (T.D.); 3 Cambridge Research Lab, Toshiba Research Europe, Cambridge CB4 0GZ, UK; pulak.isi@gmail.com

**Keywords:** semantic mapping, RGB-D SLAM, visual mapping

## Abstract

In this paper, a novel Pixel-Voxel network is proposed for dense 3D semantic mapping, which can perform dense 3D mapping while simultaneously recognizing and labelling the semantic category each point in the 3D map. In our approach, we fully leverage the advantages of different modalities. That is, the PixelNet can learn the high-level contextual information from 2D RGB images, and the VoxelNet can learn 3D geometrical shapes from the 3D point cloud. Unlike the existing architecture that fuses score maps from different modalities with equal weights, we propose a softmax weighted fusion stack that adaptively learns the varying contributions of PixelNet and VoxelNet and fuses the score maps according to their respective confidence levels. Our approach achieved competitive results on both the SUN RGB-D and NYU V2 benchmarks, while the runtime of the proposed system is boosted to around 13 Hz, enabling near-real-time performance using an i7 eight-cores PC with a single Titan X GPU.

## 1. Introduction

Real-time 3D semantic mapping is often desired in a number of robotics applications, such as localization [1,2], semantic navigation [3,4] and human-aware navigation [5]. The semantic information provided with a 3D dense map is more useful than the geometric information [6] itself in robot-human or robot-environment interaction. It enables robots to perform advanced tasks requiring high precision, such as nuclear waste classification [7] and sorting or autonomous package delivery in warehouse environments. For intelligent mobile robotics applications, extending 3D mapping to 3D semantic mapping enables robots not only to localize themselves with respect to the scene’s geometrical features, but also to simultaneously understand the higher-level semantic meaning of a complex scene.

A variety of well-known methods such as RGB-D SLAM [8], Kinect Fusion [9] and ElasticFusion [10] can generate a dense or semi-dense 3D map from RGB-D videos. However, these 3D maps contain no semantic-level understanding of the observed scenes. On the contrary, impressive results in semantic segmentation have been achieved with the advancement of convolutional neural networks (CNN). RGB [11,12,13], RGB-D [14,15,16,17] and point cloud [18,19] data have been successfully utilized for semantic segmentation. However, some of those methods are painfully slow due to their high computational demands. Thus, these methods are not yet integrated in real-time systems for robotics applications.

Compared to the well-investigated research on geometric 3D reconstruction and scene understanding, limited literature is available for 3D semantic mapping [20,21,22,23]. To date, there are no existing methods that make use of both RGB and point cloud data for semantic mapping. In this paper, we propose a dense RGB-D semantic mapping system with a Pixel-Voxel neural network, which can perform dense 3D mapping, while simultaneously recognizing and semantically labelling each point in the 3D map. The main contributions of this paper can be summarized as follows:

1. A Pixel-Voxel network consuming the RGB image and point cloud is proposed, which can obtain global context information through PixelNet while preserving accurate local shape information through VoxelNet.
2. A softmax weighted fusion stack is proposed to adaptively learn the varying contributions of different modalities. It can be inserted into a neural network to perform fusion-style end-to-end learning for arbitrary input modalities.
3. A dense 3D semantic mapping system integrating a Pixel-Voxel network with RGB-D SLAM is developed. Its runtime can be boosted to around 13 Hz using an i7 eight-core PC with Titan X GPU, which is close to the requirements of real-time applications.

The rest of this paper is organized as follows. First, the related work is reviewed in Section 2 followed by the details of the proposed methods in Section 3. The experimental results and analysis are presented in Section 4. Finally, we conclude the paper in Section 5.

## 2. Related Work

### 2.1. Dense 3D Semantic Mapping

To the best of our knowledge, the online dense 3D semantic mapping methods can be further grouped into three main sub-categories: semantic mapping based on 3D template matching [20,24], 2D/2.5D semantic segmentation [21,22,25,26,27] and RGB-D data association from multiple viewpoints [23,28,29].

The first type of methods such as SLAM++ [20] can only recognize known 3D objects in a predefined database. The approach is limited to situations where repeated and identical objects are present for semantic mapping. For the second type of methods, both approaches [21,25] adopt human-designed features with random decision forests to perform per-pixel label predictions of the incoming RGB videos. Then, all of the semantically-labelled images are associated together using visual odometry to generate the semantic map. Because of the state-of-the-art performance provided by the CNN-based scene understanding, SemanticFusion [22] integrates deconvolutional neural networks [30] with ElasticFusion [10] to obtain a real-time-capable (25 Hz) semantic mapping system. All of these three methods require fully connected CRF [31] optimization as an offline post-processing stage, i.e., the best performing semantic mapping methods are not capable of online operation. Zhao et al. [27] proposed the first system to perform simultaneous 3D mapping and pixel-wise material recognition. It integrates CRF-RNN [32] with RGB-D SLAM [8], and a post-processing optimization stage is not required. Keisuke et al. [26] proposed a real-time dense monocular CNN-SLAM method, which can perform depth prediction and semantic segmentation simultaneously from a single image using a deep neural network.

All the above methods mainly focus on semantic segmentation using a single image and perform 3D label refinement through a recursive Bayesian update using a sequence of images. However, they do not take full advantage of the associated information provided by multiple viewpoints of a scene. Yu et al. [23] proposed a data-associated recurrent neural network (DA-RNN) integrated with Kinect Fusion [9] for 3D semantic mapping. DA-RNN employs a recurrent neural network to tightly combine the information contained in multiple viewpoints of an RGB-D video stream to improve the semantic segmentation performance. Ma et al. [28] proposed a multi-view consistency layer, which can use multi-view context information for object-class segmentation from multiple RGB-D views. It utilizes the visual odometry trajectory from RGB-D SLAM [8] to wrap semantic segmentation between two viewpoints. Further, Armin et al. [29] proposed a network architecture for spatially- and temporally-coherent semantic co-segmentation and mapping of complex dynamic scenes from multiple static or moving cameras.

### 2.2. Fusion Style Semantic Segmentation

Most of the fusion-style semantic segmentation methods take advantage of both RGB and depth images. FuseNet [14] can fuse RGB and depth cues in a single encoder-decoder CNN architecture for RGB-D semantic segmentation. The long short-term memorized context fusion (LSTM-CF) network [15] fuses contextual information from multiple channels of RGB and depth images through stacking of several convolution layers and a long short-term memory layer. FuseNet normalizes the depth value into the interval of [0,255] to have the same spatial range as colour images, while the LSTM-CF network encodes depth to a horizontal, height, angle (HHA) image to obtain three channels as the colour image. The HHA representation can improve the depth-based semantic segmentation; however, the HHA representation requires a high computational cost and hence cannot be performed in real time. Spatio-temporal data-driven pooling (STD2P) [33] involves a novel superpixel-based multi-view convolutional neural network for RGB-D semantic segmentation, which uses the spatio-temporal pooling layer to aggregate information over space and time. Locality-sensitive deconvolution networks (LS-DeconvNets) [16] involve a locality-sensitive DeconvNet to refine the boundary segmentation and also a gated fusion layer for combining modalities (RGB and HHA); however the number of input modalities is limited to two. Lin et al. [17] introduced a cascaded feature network (CFN) with a context-aware receptive field (CaRF) with a better control on the relevant contextual information of the learned features for RGB-D semantic segmentation. All of the above RGB-D fusion networks treat the depth image similarly to an RGB image using a CNN with a max-pooling layer. However, this also makes the depth image lose shape information. In contrast, the 3D point cloud should have more 3D geometry information compared to the depth image. We believe there should be the potential to combine RGB and point cloud data for semantic segmentation. The forerunner work PointNet [18] provides a unified architecture for both classification and segmentation, which consumes the raw unordered point clouds as input. PointNet only employs a single max-pooling layer to generate the global feature, which describes the original input clouds; thus, it does not capture the local structures induced by the 3D metric space points live in. The improved version PointNet++ [19] is a hierarchical neural network that applies PointNet recursively on a nested partitioning of the input point set, which can learn local features with increasing contextual scales.

### 2.3. Discussion

For the task of semantic segmentation, conventional CNN-based methods have struggled with the balance between global and local information. The global context information can alleviate the local ambiguities to improve the recognition performance, while local information is crucial to obtain accurate per-pixel accuracy, i.e., shape information. How to increase the receptive field to get more global context information, while preserving a high resolution feature map, is still an open problem.

Processing the depth image in a similar manner to the RGB image using CNN with max-pooling cannot preserve all the local geometry information. Compared to RGB and RGB-D data, a 3D point cloud can provide rich spatial information. For example, in PointNet [18], a single fully-connected multi-layer network followed by a single global max-pooling layer are used for semantic segmentation of a point cloud. The resolution does not decrease, and it can keep the original spatial information of the data. However, these methods lack the context information because of the usage of a single global max-pooling layer. Intuitively, combining RGB-based and point cloud-based networks together can alleviate each of their drawbacks and leverage each of their advantages. The RGB image can provide global context information as a supplement for point cloud segmentation, while the point cloud can help refine the boundary shape for RGB segmentation.

Moreover, during RGB-D mapping, both the RGB image and point cloud can be obtained directly from an RGB-D camera, which is easily available and enables a potential combination for semantic mapping. This motivated us to utilize a Pixel-Voxel neural network for dense RGB-D semantic mapping.

In addition, the networks in [11,14,15,17] simply fuse the score maps from different modalities using equal weights. The gated fusion in LS-DeconvNets [16] is limited to fusion of the features from (at most) two modalities. However, each modality should have different contributions in different situations for different categories. Therefore, in this paper, a softmax weighted fusion stack is proposed for adaptively learning the varying contribution of each modality.

## 3. Proposed Method

### 3.1. Overview

**Figure 1:**
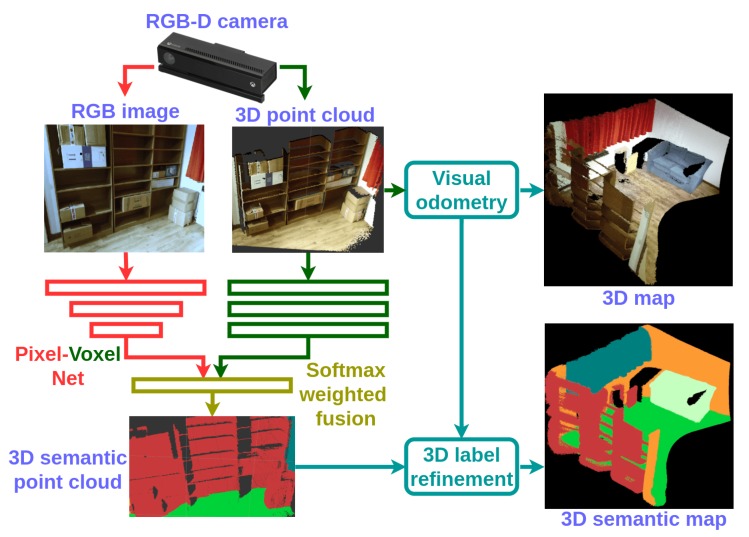
The pipeline of the proposed dense RGB-D semantic mapping with the Pixel-Voxel neural network. The RGB image and 3D point cloud are obtained from an RGB-D camera, Kinect V2. The RGB and point cloud data-pair of each key-frame is fed into the Pixel-Voxel network for semantic segmentation. The semantically-labelled point clouds are then combined incrementally through the visual odometry of RGB-D SLAM. The label probability of each voxel is further refined by a recursive Bayesian update. Finally, the dense 3D semantic map is generated.

**Figure 2:**
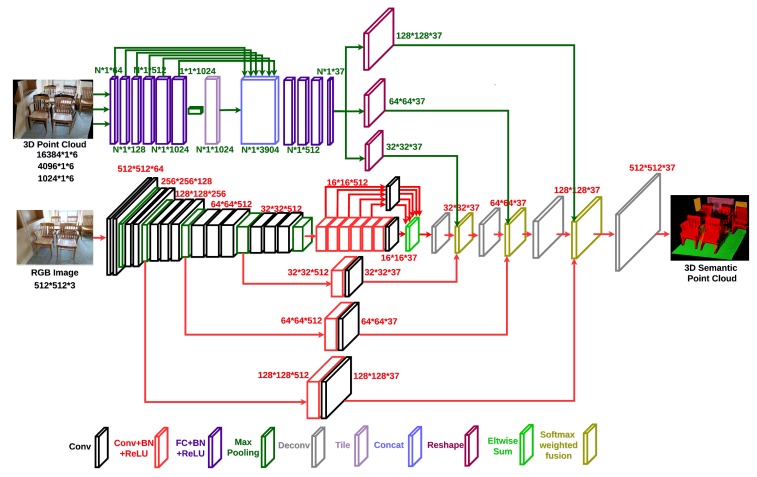
The architecture of the proposed Pixel-Voxel network. The proposed architecture consists of two parallel feed-forward sub-networks: PixelNet and VoxelNet. The PixelNet is comprised of three building blocks: truncated CNN, context stack and skip architecture. The VoxelNet is composed of the following blocks: fully-connected stacks, local and global information combination stack and reshape layer. It obtains global context information through PixelNet while preserving accurate local shape information through VoxelNet. The enlarged architecture of the softmax weighted fusion stack can be found in Figure 3. It can fuse the score maps from PixelNet and VoxelNet according to their respective confidence at different resolutions.

The pipeline of the proposed dense RGB-D semantic mapping with a Pixel-Voxel neural network is illustrated in Figure 1. The input RGB image and point cloud pairs of each key-frame are fed into the Pixel-Voxel network. The architecture of the proposed network is displayed in Figure 2. The output of the network—a semantically-labelled point cloud—is combined incrementally according to the visual odometry of RGB-D SLAM. The label probability of each voxel is refined by a recursive Bayesian update. Finally, the dense 3D semantic map is generated. Note that in our current architecture, a voxel consists of just a single 3D point.

### 3.2. Pixel Neural Network

The sub-network PixelNet is comprised of three units: truncated CNN, a context stack similar to [34] and the skip architecture. The input of PixelNet is an RGB image. For the truncated CNN, VGG-16 (http://www.robots.ox.ac.uk/~vgg/research/very_deep/) or ResNe (https://github.com/KaimingHe/deep-residual-networks) (truncated after pool5), pre-trained on ImageNet (http://www.image-net.org/challenges/LSVRC/), can be employed as a baseline. After the truncated CNN, the resolution of the feature maps is decreased 32-times compared with the input image; thus, it drops a significant amount of shape information, which is recovered utilizing the VoxelNet sub-network.

Note that the receptive fields after the pool5 layer of VGG-16 are of dimension 212×212, which is not large enough to cover the whole 512×512 input image. Therefore, a context-stack, composed of a chain of 6 layers of 5×5×512 convolution stacks [Conv+BN+ReLU], is concatenated on the top of a pre-trained truncated VGG-16 network. The context stack can expand the receptive field progressively, as shown in Figure 4, to cover all the elements in the current feature map (the whole original image). The receptive field of the context stack can be described as:

(1) $${\mathrm{RF}}_{j}={\mathrm{RF}}_{j-1}+\left(k_{j}-1\right)\times \prod_{i=0}^{j-1}S_{i},j\;\in \left[1,n\right]$$

where ${\mathrm{RF}}_{j}$ and $k_{j}$ are the receptive field and kernel size of the *j*-th context stack, $S_{i}$ refers to the stride of the *i*-th context stack, ${\mathrm{RF}}_{0}$ and $S_{0}$ are the receptive field and stride product before the first context stack and n=6 is the number of context stacks. In addition, the score maps of all the context stacks are fused together to aggregate multi-scale context information. Notice that the spatial dimensionality of the feature maps in a context stack is unchanged.

The skip architecture consists of 3 skip stacks [Conv+BN+ReLU+Conv(score)] following pool2, pool3 and pool4 separately. In order to prevent the network training from divergence, conventionally, a smaller learning rate is adopted for the skip architecture during training (similar to [11]). We utilize batch normalization, which stabilizes the back-propagated error signals; thus, a bigger learning rate (0.01) can be employed for training. The skip architecture retains the low-level features of the RGB image.

### 3.3. Voxel Neural Network

The input of VoxelNet is a point cloud, which is represented as a set of 3D points $\left\{p_{i}\;|\;i=1,2\ldots ,n\;\right\}$ stored in a vector of length n×6, where *n* is the number of points and $p_{i}$ is a 6-dimensional vector containing position information ${\left(X,Y,Z\right)}^{T}$ in the world coordinates and pixel colour information ${\left(R,G,B\right)}^{T}$. Inspired by PointNet [18], we also use max pooling as an invariant function. The max-pooling operation obtains the global feature from all the points, which are concatenated with the pixel features to predict point-wise semantic labels. The higher dimensional feature representation for each point of the subnetwork can be summarized by the following equation.

(2) $$\begin{array}{c} \left[F_{global}^{1}\ldots F_{global}^{n}\right]=T\left(M\left([f_{mlp}^{k}\left(p_{1}\right)\ldots f_{mlp}^{k}\left(p_{n}\right)]\right)\right) \end{array}$$

Here, $f_{mlp}$ is the multi-layer perception network, i.e., FC+BN+ReLU, and *k* is the number of multi-layer perception networks before max pooling. Each point shares the same set of fully-connected weights. M is the max pooling operation with kernel size n×1, and T is the tile operation, which restores the shape of the feature map from 1×1 to n×1.

The output $\left[F_{global}^{1}\ldots F_{global}^{n}\right]$ is the global feature map of the input set. This is fed to the per-point feature of the multi-layer perception network to concatenate the global and local information.

(3) $$\left[F_{concat}^{1}\ldots F_{concat}^{n}\right]=Concat\left(\left[F_{global}^{1}\ldots F_{global}^{n}\right],\ldots \left[f_{mlp}^{i}\left(p_{1}\right)\ldots f_{mlp}^{i}\left(p_{n}\right)\right]\right),i\;\in \left[1,k\right]$$

Then, the new per-point features are extracted though the multi-layer perception network using the combined global and local point features as:

(4) $$F_{h\times w}^{1\ldots n}=R\left([f_{mlp}^{m}\left(F_{concat}^{1}\right)\ldots f_{mlp}^{m}\left(F_{concat}^{n}\right)]\right)$$

where *m* is the last multi-layer perception network and R is the reshape operation, which transforms the shape of the score map from n×1 to h×w through back-projection (It is worth noting that the distortions are incorporated during the projection to pixel coordinates):

(5) $$d_{u,v}\;\left[\begin{array}{c} u\\ v\\ 1 \end{array}\right]=\left[\begin{array}{c} f_{x}\;s\;c_{x}\\ 0\;f_{y}\;c_{y}\\ 0\;\;0\;\;1 \end{array}\right]\;\left[\begin{array}{c} X\\ Y\\ Z \end{array}\right]$$

where $f_{x},f_{y}$ are the focal lengths, ($c_{x},c_{y}$) is the principal point offset, *s* is the axis skew and (u,v) is the pixel position in the image plane. Here, the radial distortion had been incorporated during the projection to the pixel coordinates. In detail, the feature of the point cloud in (X,Y,Z) can be transformed to the position (u,v) in the image plane, so the score map of VoxelNet can be fused with the score map of PixelNet.

The spatial dimensionality of the features is the same as that of the input data in VoxelNet, so it can preserve all the original shape information. However, if only a single max pooling layer is adopted to generate the global feature, it will drop significant context information from the input point cloud.

### 3.4. Softmax Weighed Fusion

**Figure 3:**
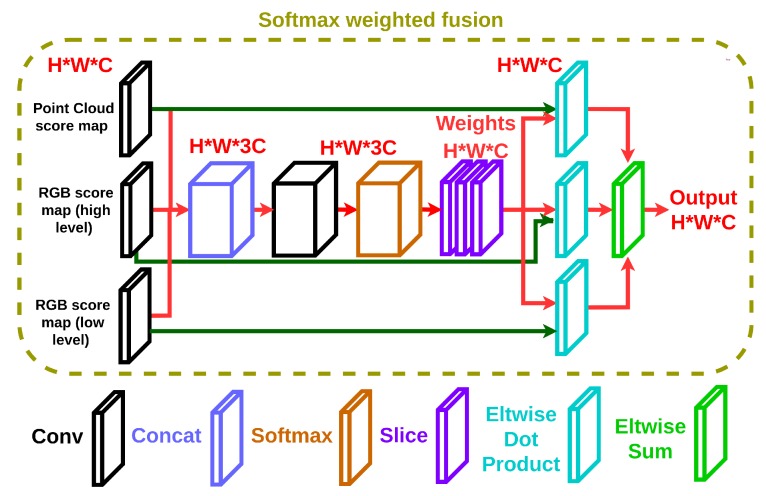
The architecture of the softmax weighted fusion stack. H, W, C are the hight, width and channel number of the feature map. The convolution operation can learn the correlations of the multiple score maps from different modalities to obtain the weight/confidence of each modality.

**Figure 4:**
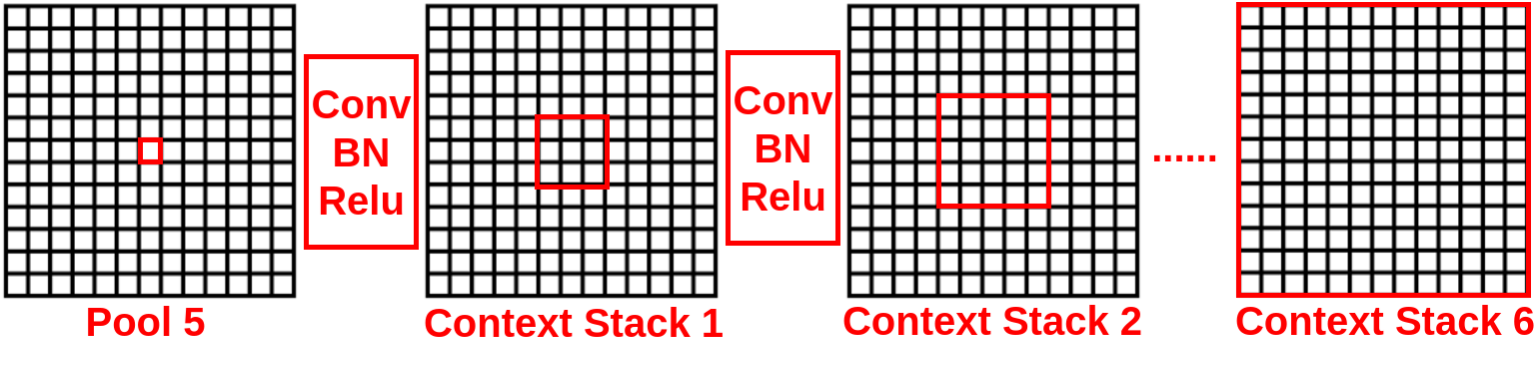
The receptive field (the area of red square) of the context stack is progressively extended to cover all the elements in the feature map.

In contrast to the conventional methods, which simply fuse score maps from different modalities using equal weights, a softmax weighted fusion stack, as shown in Figure 3, is designed to learn the varying contribution of each modality in different situations for different categories. To be precise, let us define the score maps by $F^{1},F^{2}\ldots F^{n}\in R^{c\times h\times w}$, generated from *n* different modalities, where *c* is the number of categories and h×w are the dimensions of the score map. Then, the fusion score map $F_{fused}\in R^{n\cdot c\times h\times w}$ can be written as:

(6) $$F_{fused}=C\left(\left[F^{1},F^{2}\ldots F^{n}\right]\right)⊛W_{conv}$$

where ⊛ is the convolution operation, C is the concatenation operation and $W_{conv}\in R^{n\cdot c\times n\cdot c\times 1\times 1}$ are the weights of the convolution. The convolution operation learns the correlations of the multiple score maps from *n* different modalities. The channel values of $F_{fused}$ are further normalized into the interval [0,1] according to the softmax operation. Then, the weights of the score map are obtained through a slice operation as:

(7) $$W^{1},W^{2}\ldots W^{n}=S\left[\mathrm{softmax}(F_{fused})\right]$$

where S is the slice operation, $\mathrm{softmax}\left(x\right)=\frac{\exp(x)}{\sum_{i=1}^{n\cdot c}\exp\left(x_{i}\right)}$ and $W^{1},W^{2}\ldots W^{n}\in R^{c\times h\times w}$ are the corresponding weights of the score maps. The weights signify the confidence of each model. The weighted fusion score map $F_{score}\in R^{c\times h\times w}$ can be written as:

(8) $$F_{score}=\sum_{j=1}^{n}F^{j}⊙W^{j},$$

where ⊙ is the element-wise multiplication operation, $\sum_{j=1}^{n\cdot c}W^{j}=1$ and $1\in R^{h\times w}$.

For our problem, the three score maps from PixelNet and VoxelNet are fused together according to their respective confidence levels. Note that the proposed weighted fusion stack can fuse the score maps of an arbitrary number of modalities. Moreover, it can be easily inserted into a neural network that requires fusion of multiple modalities and can be trained end-to-end. Thus, it can potentially be applied to many other similar problems.

### 3.5. Class-Weighted Loss Function

In most of the datasets for semantic segmentation, we observe highly imbalanced class distributions. Thus, focusing more on the rare classes to boost their recognition accuracy can improve the average recognition performance significantly, while overall recognition performance might decrease a little. We adopt the class-weighted negative log-likelihood as the loss function:

(9) $$\mathrm{loss}=-\sum_{i\in \Theta }\left(1_{y_{i}=j}\right)2^{\left\lceil \log_{10}\left(\delta /p_{j}\right)\right\rceil }\cdot \log L\left(\mathrm{softmax}\left(F_{i}\right),y_{i}\right)$$

where Θ are the training data, L is the likelihood function, $F_{i}$ is the final score map, $y_{i}$ refers to the one-hot training label. $1_{y_{i}=j}$ is a function that returns 1 if $y_{i}=j$, or 0 otherwise. $p_{j}$ is the occurrence frequency of class *j*, and $2^{\left\lceil \log_{10}\left(\delta /p_{j}\right)\right\rceil }$ is the weight of class *j*. δ is the threshold of frequency criteria for the rare class. ⌈⌉ is the ceiling operation. This will force the network to assign a higher weight to rare classes. The value of δ is set to 2.5% following the 85%–15% rule described in [35], i.e., the frequency sum of all the rare classes is 15%.

### 3.6. RGB-D Mapping and 3D Label Refinement

RGB-D SLAM [8] is adopted for dense 3D mapping. Its visual odometry can provide the transformation information between two adjacent semantically-labelled point clouds. It is then used for generating a global semantic map and enabling incremental semantic label fusion.

After obtaining the semantically-labelled point clouds from different viewpoints, label hypotheses are fused by a recursive Bayesian update to refine the 3D semantic map. Each voxel in the semantic point cloud stores both the label value and the corresponding discrete probability. The voxels from different viewpoints can be transformed to the same coordinate through the visual odometry of RGB-D SLAM. Then, the voxel’s label probability distribution is updated by means of a recursive Bayesian update as:

(10) $$P\left(x=l_{i}|I_{1,\ldots ,k}\right)=\frac{1}{Z}P\left(x=l_{i}|I_{1,\ldots ,k-1}\right)P\left(x=l_{i}|I_{k}\right)$$

where $l_{i}$ is the label prediction, $I_{k}$ is the *k*-th frame and *Z* is the normalizing constant. The label refinement is applied to all label probabilities of each voxel to generate a proper distribution.

## 4. Experiments

We evaluate the proposed Pixel-Voxel network using two popular indoor scene datasets, i.e., the SUN RGB-D (http://rgbd.cs.princeton.edu/) and NYU V2 (https://cs.nyu.edu/~silberman/datasets/nyu_depth_v2.html) datasets. The former is used to evaluate the semantic segmentation on a single frame, while the latter provides raw RGB-D sequences, which can be used for the semantic segmentation evaluation on multiple frames.

The SUN RGB-D dataset contains 5285 synchronized RGB-D image pairs for training/validation and 5050 synchronized RGB-D image pairs for testing. The RGB-D image pairs with different resolutions are captured by 4 different RGB-D sensors: Kinect V1, Kinect V2, Xtion and RealSense. The task is to segment 37 indoor scene classes such as table, chair, sofa, window, door, etc. Pixel-wise annotations are available in these datasets. However, the extremely unbalanced distribution of class instances makes the task very challenging. The rareness frequency threshold is set to 2.5% in the class-weighted loss function following the 85–15% rule.

The NYU V2 dataset provides synchronized 1449 pixel-wise annotated RGB-D image pairs captured by Kinect V1, which includes 795 frames for training/validation and 654 frames for testing. The task is to segment 13 classes similar to the SUN RGB-D dataset in an indoor scene. Comparing with the other larger RGB-D datasets, the NYU V2 dataset provides raw RGB-D videos rather than discrete single frames. Therefore, using the odometry of RGB-D SLAM, the semantic segmentation based on multiple frames can be evaluated for the dense semantic mapping.

### 4.1. Data Augmentation and Preprocessing

For the PixelNet training, all the RGB images are resized to the same resolution 512×512 through bilateral filtering. We randomly flip the RGB image horizontally and rescale the image slightly to augment the RGB training data.

For the VoxelNet training, there is still no available large-scale ready-made 3D point cloud dataset. We generated the point cloud using the RGB-D image pairs and the corresponding intrinsic parameters of the camera through back-projection, e.g., Equation (5) for the SUN RGB-D and NYU V2 datasets. Following [14], 514 training and 558 testing RGB-D image pairs containing invalid values, which might lead to incorrect supervision during training, are excluded from the SUN RGB-D dataset. We also randomly flip the 3D point cloud horizontally to augment the training data. There is huge computational complexity if the original point clouds are used for VoxelNet training. Therefore, we uniformly down-sample the original point cloud to a sparse point cloud in 3 different scales. The numbers of points in these sparse point clouds are 16,384, 4096 and 1024, respectively.

### 4.2. Network Training

The whole training process can be divided into 3 stages: PixelNet training, VoxelNet training and Pixel-Voxel network training. Firstly, PixelNet and VoxelNet are each trained separately. Then, the pre-trained weights are inherited for the Pixel-Voxel network training.

All the networks are trained using stochastic gradient descent with momentum. The batch size is set to 10, the momentum fixed to 0.9 and the weight decay fixed to 0.0005. The new parameters are randomly initialized from a Gaussian distribution with variance $10^{-2}$. The step learning policy is adopted for PixelNet training, and the polynomial learning policy is adopted for PixelNet and Pixel-Voxel Network training. The learning rate is initialized to $10^{-3}$, and the learning rate of the newly-initialized parameters is set to 10-times higher than that of the pre-trained parameters. Because there are 3 softmax weighed fusion stacks, 3 rounds of fine-tuning are required during the Pixel-Voxel network training.

### 4.3. Overall Performance

Following [11], three standard performance metrics for semantic segmentation are used for the evaluation: pixel accuracy, mean accuracy and mean intersection over union (IoU). The three metrics are defined as:

- Pixel accuracy: $\sum_{i}n_{ii}/\sum_{i}t_{i}$
- Mean accuracy: $\left(1/n_{cl}\right)\sum_{i}n_{ii}/t_{i}$
- Mean IoU: $\left(1/n_{cl}\right)\sum_{i}n_{ii}/\left(t_{i}+\sum_{j}n_{ji}-n_{ii}\right)$

where $n_{cl}$ is the number of classes, $n_{ij}$ is the number of pixels of class *i* classified as class *j* and $t_{i}=\sum_{j}n_{ij}$ is the total number of pixels belonging to class *i*.

In the experiment on the SUN RGB-D dataset, the performance of the Pixel-Voxel network and all the baselines are evaluated on a single frame. In the second experiment, the results are obtained by fusing multiple frames (provided by the raw data). To be more specific, visual odometry is employed to associate the pixels in consecutive frames, and then, a Bayesian-update-based 3D refinement is used to fuse all predictions. Similar strategies are used in the baseline methods, i.e., Hermans et al. [21], SemanticFusion [22] and Ma et al. [28].

**Figure 5:**
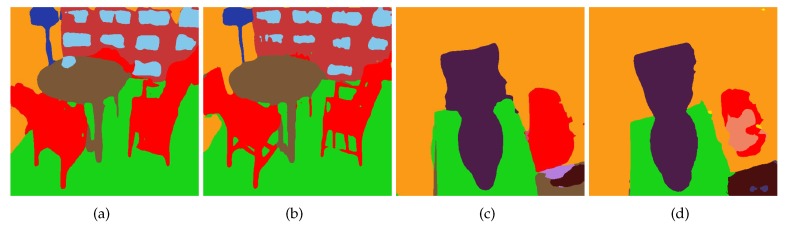
(**a**,**c**) are the coarse predictions from PixelNet, and (**b**,**d**) are the predictions after combining VoxelNet with PixelNet. It can be seen that the boundary shape is more accurate after the VoxelNet refinement. The colour palette can be found in Figure 6.

**Figure 6:**
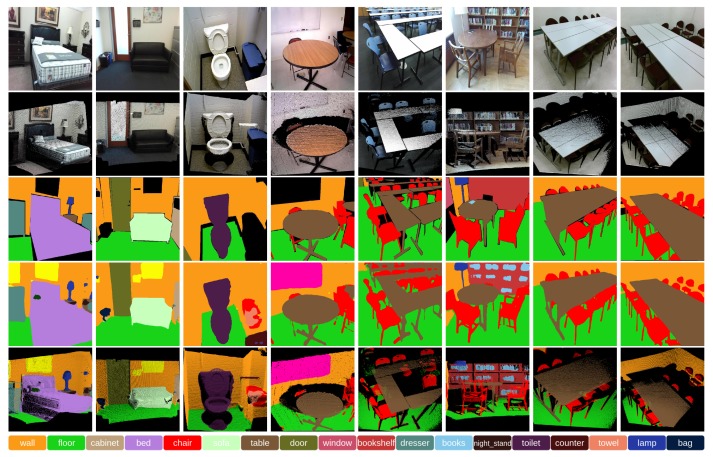
Qualitative results (best viewed in colour) for the Pixel-Voxel network on the SUN RGB-D dataset. For different scenes in each row, the following images are displayed: RGB image (Row 1), 3D point cloud (Row 2), ground truth image (Row 3), 2D semantic image (Row 4) and 3D semantic point cloud (Row 5). The Pixel-Voxel network produces results with accurate boundary shape such as the shape of the bed, toilet and especially the legs of the furniture.

From Figure 5 and Figure 6, it is clear that after combining VoxelNet with PixelNet, the edge prediction can be improved significantly. Preserving 3D shape information through VoxelNet, the results have accurate boundaries, such as the shape of the bed, toilet and especially the legs of the furniture.

The comparison of overall performance on the SUN RGB-D and NYU V2 datasets are shown in Table 1 and Table 2. The class-wise accuracy on the SUN RGB-D and NYU V2 datasets are shown in Table 3 and Table 4. The class-wise IoU of the Pixel-Voxel network is also provided. For the SUN RGB-D dataset, we achieved 79.04% for overall pixel accuracy, 57.65% for mean accuracy and 44.24% for mean IoU. After combining VoxelNet edge refinement, the pixel accuracy increased slightly from 77.25%–77.82% for VGG-16 and from 78.30%–78.76% for ResNet101, while the mean accuracy shows a significant increase from 49.33%–53.86% for VGG-16 and from 54.22%–56.81% for ResNet101. For the NYU V2 dataset, we achieved an overall pixel accuracy of 82.53%, a mean accuracy of 74.43% and a mean IoU of 59.30%. After combining VoxelNet edge refinement, the overall accuracy increases slightly from 80.74%–81.50% for VGG-16 and from 81.63%–82.22% for ResNet101, while the mean accuracy shows a significant increase from 70.23%–72.25% for VGG-16 and from 72.18%–73.64% for ResNet101.

Modelling the global context information and simultaneously preserving the local shape information are the two key problems in CNN-based semantic segmentation. The main idea of Pixel-Voxel net is to leverage the advantages of two complementary modalities, to extract high-level context features from RGB and fuse them with low-level geometric features from the point cloud. The improvement can be attributed to three parts: the hierarchical convolutional stack in PixelNet, the boundary refinement by VoxelNet and the softmax weighted fusion stack. First, the hierarchical convolutional stack can learn the high-level contextual information through an incrementally-enlarged receptive field. As shown in Table 1 and Table 2, the standalone PixelNet can achieve a very competitive performance. Second, the proposed VoxelNet can refine the 3D object boundaries through learning the low-level geometrical features from the point clouds. As shown in Figure 5, the objects have finer boundaries after combining with VoxelNet. As shown in Table 1 and Table 2, the quantitative performance improves significantly through 3D-based shape refinement from VoxelNet. Third, the proposed softmax fusion layer can adaptively learn the confidence of each modality. As a result, the predictions from different modalities can be fused more effectively. As shown in Table 1 and Table 2, the quantitative results also increase slightly through the softmax fusion stack. Note that the overall accuracy cannot be improved significantly, as pixels/voxels on the object edge only occupy a very small percentage of the whole pixels/voxels. However, the mean accuracy experiences a substantial improvement due to the increased accuracy on rare classes, for which the edge pixels occupy a relatively large percentage of all pixels.

Most state-of-the-art methods employ multi-scale CRF or a 2D/3D graph to refine the object boundaries. Their main limitation is slowness because of the excessive usage of multi-resolution high computational CRF or graph optimization. Although their performance is slightly better than ours, these methods are unlikely to be applied to real-time robotics applications. Our method can preserve the fine boundary shape through learning the low-level features from 3D geometry data. There is no computational optimization in the Pixel-Voxel network, so it is faster than most state-of-the-art methods.

### 4.4. Dense RGB-D Semantic Mapping

The dense RGB-D semantic mapping system is implemented under the ROS (http://www.ros.org/) framework and executed on a desktop with i7-6800k (3.4 Hz) 8-core CPU and NVIDIA TITAN X GPU (12G). Kinect V2 is used to obtain the RGB images and point clouds. IAI Kinect2 package2 (https://github.com/code-iai/iaikinect2/) is employed to interface with ROS and calibrate with the Kinect2 cameras. The Pixel-Voxel network is implemented using the Caffe (http://caffe.berkeleyvision.org/) toolbox. The network is trained on a TITAN X GPU, accelerated by CUDA and CUDNN.

**Figure 7:**
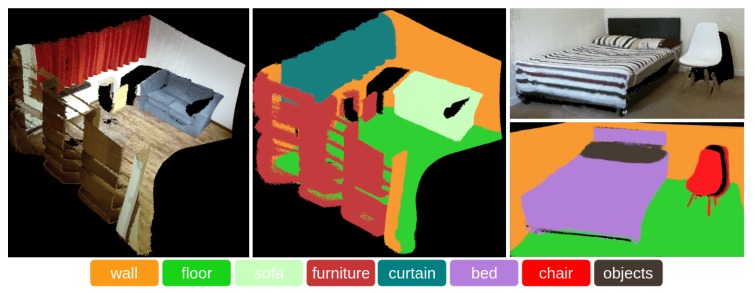
The dense 3D map and dense 3D semantic map (best viewed in colour) of a living room and bedroom.

The system with a pre-trained network was also tested in a real-world environment, e.g., a living room and bedroom containing a curtain, bed, etc., as shown in Figure 7. It can be seen that most of the point clouds are correctly segmented, and the results have accurate boundaries, but there are still some points on the boundary with wrongly-assigned labels. Some error predictions are caused by upsampling the data through a bilateral filter to the same size as the Kinect V2 data. Furthermore, this network was trained using the SUN RGB-D and NYU V2 datasets, but was tested using the real-world data. Therefore, some errors occur due to illumination variances, category variances, etc. In addition, the noise of the Kinect V2 also causes some errors in predictions.

Using the quad high definition (QHD) data from Kinect2, the runtime performances of our system are 5.68 Hz (VGG16) and 3.23 Hz (ResNet101) when the RGB is resized to 512×512 and the point cloud is down-sampled to three scales, 16,384 × 1, 4096 × 1 and 1024 × 1. During real-time RGB-D mapping, only a few key-frames are used for mapping. Most of the frames are abandoned because of the small variance between two consecutive frames. It is not necessary to segment all the frames in the sequence, but only the key-frames. As mentioned in [21], the 5-Hz runtime performance is nearly sufficient for real-time dense 3D semantic mapping. It is worth noting that the running time can be boosted to 13.33 Hz (VGG16) and 9.01 Hz (ResNet101) using half-sized data with a corresponding decline in segmentation performance. Thus, there is a trade-off between performance requirement and time consumption. The inference running time of Pixel-Voxel Net using different sizes of data can be found in Table 5, and the corresponding decline in performance can be found in Table 6.

## 5. Conclusions

This paper introduced an end-to-end discriminative Pixel-Voxel network for dense 3D semantic mapping. The hierarchical convolutional stack structure in PixelNet can model the high-level contextual information through an incrementally-enlarged receptive field, while the VoxelNet learns geometrical shapes via a non-linear feature transform in order to identify 3D objects with fine object boundaries. More importantly, an adaptive fusion layer, i.e., softmax fusion, can learn the probabilistic confidences in order to fuse features from RGB and depth (3D) modalities in the non-linear fashion. We achieved competitive performance on the SUN RGB-D benchmark (pixel acc.: 79.04%, mean acc.: 57.65% and mean IoU: 44.24%) and NYU V2 benchmark (pixel acc.: 82.53%, mean acc.: 74.43% and mean IoU: 59.30%). Our method is fully parametric without running time optimizations. Consequently, a straightforward inference is used for deployment, which guarantees near-real-time performance. Our method is faster than most state-of-the-art methods (up to around 13 Hz using an i7 eight-core PC with Titan X GPU) and can be integrated into a SLAM system for near-real-time application in robotics.

For future work, we will investigate the possibility of applying the proposed VoxelNet for semantic segmentation [40] with 3D LiDAR data, where only 3D geometric data are available. Moreover, and we will investigate adopting the proposed semantic mapping method to domestic robot navigation and manipulation tasks. The source code will be published upon acceptance. A real-time demo can be found on the author’s Youtube channel (https://youtu.be/UbmfGsAHszc).

## Figures and Tables

**Table 1:** Comparison of the overall performance on the SUN RGB-D dataset. Some results are copied from [12]. The best performance among the compared methods is marked as bold.

Methods	Pixel Acc.	Mean Acc.	Mean IoU
FCN [11]	68.18%	38.41%	27.39%
DeconvNet [30]	66.13%	33.28%	22.57%
SegNet [12]	72.63%	44.76%	31.84%
DeepLab [13]	71.90%	42.21%	32.08%
Context-CRF [36]	78.4%	53.4%	42.3%
LSTM-CF [15] (RGB-D)	-	48.1%	-
FuseNet [14] (RGB-D)	76.27%	48.30%	37.29%
LS-DeconvNets (RGB-D) [16]	-	58.00%	-
RefineNet-Res101 [37]	80.4%	57.8%	45.7%
RefineNet-Res152 [37]	**80.6%**	**58.5%**	45.9%
CFN (VGG-16, RGB-D) [17]	-	-	42.5%
CFN (RefineNet-152, RGB-D) [17]	-	-	**48.1%**
Pixel Net (VGG-16)	77.25%	49.33%	38.26%
Pixel Net (ResNet101)	78.30%	54.22%	41.73%
Pixel-Voxel Net (VGG-16, without fusion)	77.82%	53.86%	41.33%
Pixel-Voxel Net (ResNet101, without fusion)	78.76%	56.81%	43.59%
Pixel-Voxel Net (VGG-16)	78.14%	54.79%	42.11%
Pixel-Voxel Net (ResNet101)	79.04%	57.65%	44.24%

**Table 2:** Comparison of overall performance on the NYU V2 dataset. Some results are copied from [28]. The methods with † take advantage of the data from multiple views. The best performance among the compared methods is marked as bold.

Methods	Pixel Acc.	Mean Acc.	Mean IoU
Hermans et al. [21] (RGB-D) †	54.3%	48.0%	-
SemanticFusion [22] †	67.9%	59.2%	-
SceneNet [38]	67.2%	52.5%	-
Eigen et al. [39] (RGB-D)	75.4%	66.9%	52.6%
FuseNet [14] (RGB-D)	75.8%	66.2%	54.2%
Ma et al. [28] (RGB-D) †	79.13%	70.59%	59.07%
Pixel Net (VGG-16) †	80.74%	70.23%	55.92%
Pixel Net (ResNet101) †	81.63%	72.18%	57.78%
Pixel-Voxel Net (VGG-16, without fusion) †	81.50%	72.25%	57.69%
Pixel-Voxel Net (ResNet101, without fusion) †	82.22%	73.64%	58.71%
Pixel-Voxel Net (VGG-16) †	81.85%	73.21%	58.54%
Pixel-Voxel Net (ResNet101) †	**82.53%**	**74.43%**	**59.30%**

**Table 3:** Comparison of the class-wise accuracy on the SUN RGB-D dataset. Some of the methods in Table 1 do not provide the class-wise accuracy; hence, they are omitted here. The class-wise IoU of the Pixel-Voxel network (PVNet) is also provided. LS, locality-sensitive. The best performance among the compared methods is marked as bold.

**Category**	**Wall**	**Floor**	**Cabinet**	**Bed**	**Chair**	**Sofa**	**Table**	**Door**	**Window**	**Bookshelf**	**Picture**	**Counter**	**Blinds**
SegNet [12]	83.42%	93.43%	63.37%	73.18%	75.92%	59.57%	64.18%	52.50%	57.51%	42.05%	56.17%	37.66%	40.29%
LSTM-CF [15]	74.9%	82.3%	47.3%	62.1%	67.7%	55.5%	57.8%	45.6%	52.8%	43.1%	56.7%	39.4%	48.6%
FuseNet [14]	90.20%	94.91%	61.81%	77.10%	78.62%	66.49%	65.44%	46.51%	62.44%	34.94%	67.39%	40.37%	43.48%
LS-DeconvNets [16]	**91.9%**	94.7%	61.6%	**82.2%**	**87.5%**	62.8%	**68.3%**	47.9%	**68.0%**	48.4%	**69.1%**	49.4%	51.3%
PVNet (VGG16)	90.28%	93.21%	66.87%	75.31%	85.45%	**67.37%**	64.81%	58.62%	63.58%	54.54%	64.76%	**51.87%**	**59.23%**
PVNet (ResNet101)	89.19%	**94.94%**	**69.36%**	79.11%	85.70%	66.09%	60.59%	**62.22%**	66.59%	**58.34%**	66.39%	50.56%	53.65%
PVNet (VGG16)${}_{IoU}$	76.07%	87.20%	50.66%	68.23%	64.98%	54.17%	**46.07%**	44.83%	46.50%	41.31%	**48.94%**	41.19%	**39.95%**
PVNet (ResNet101)${}_{IoU}$	**77.41%**	**87.78%**	**53.44%**	**71.16%**	**66.76%**	**54.61%**	44.46%	**45.19%**	**48.23%**	**41.79%**	46.78%	**41.39%**	35.95%
**Category**	**Desk**	**Shelves**	**Curtain**	**Dresser**	**Pillow**	**Mirror**	**Floor_Mat**	**Clothes**	**Ceiling**	**Books**	**Fridge**	**TV**	**Paper**
SegNet [12]	11.92%	11.45%	66.56%	52.73%	43.80%	26.30%	0.00%	34.31%	74.11%	53.77%	29.85%	33.76%	22.73%
LSTM-CF [15]	**37.3%**	9.6%	63.4%	35.0%	45.8%	44.5%	0.0%	28.4%	68.0%	47.9%	61.5%	52.1%	36.4%
FuseNet [14]	25.63%	20.28%	65.94%	44.03%	54.28%	52.47%	0.00%	25.89%	84.77%	45.23%	34.52%	34.83%	24.08%
LS-DeconvNets [16]	35.0%	24.0%	**68.7%**	60.5%	**66.5%**	57.6%	0.00%	**44.4%**	**88.8%**	**61.5%**	51.4%	71.7%	37.3%
PVNet (VGG16)	32.05%	23.09%	62.49%	62.13%	54.97%	50.60%	**0.59%**	35.35%	57.78%	41.75%	55.43%	67.60%	35.34%
PVNet (ResNet101)	32.49%	**27.37%**	68.33%	**69.41%**	56.96%	**57.94%**	0.00%	36.45%	68.77%	42.02%	**63.05%**	**72.47%**	**38.11%**
PVNet (VGG16)${}_{IoU}$	**26.05%**	12.05%	50.52%	47.43%	36.35%	36.44%	**0.59%**	20.56%	53.61%	28.04%	41.23%	57.36%	24.13%
PVNet (ResNet101)${}_{IoU}$	25.30%	**16.86%**	**53.09%**	**50.83%**	**38.16%**	**42.29%**	0.00%	**22.28%**	**63.39%**	**29.21%**	**48.47%**	**60.46%**	**25.20%**
**Category**	**Towel**	**Shower_Curtain**	**Box**	**Whiteboard**	**Person**	**Night_Stand**	**Toilet**	**Sink**	**Lamp**	**Bathtub**	**Bag**	**Mean**	**-**
SegNet [12]	19.83%	0.03%	23.14%	60.25%	27.27%	29.88%	76.00%	58.10%	35.27%	48.86%	16.76%	31.84%	-
LSTM-CF [15]	36.7%	0.0%	38.1%	48.1%	**72.6%**	36.4%	68.8%	67.9%	58.0%	65.6%	23.6%	48.1%	-
FuseNet [14]	21.05%	**8.82%**	21.94%	57.45%	19.06%	37.15%	76.77%	68.11%	49.31%	73.23%	12.62%	48.30%	-
LS-DeconvNets [16]	**51.4%**	2.9%	**46.0%**	54.2%	49.1%	**44.6%**	**82.2%**	**74.2%**	**64.7%**	**77.0%**	**47.6%**	**58.0%**	-
PVNet (VGG16)	41.12%	4.59%	40.33%	66.56%	60.51%	33.21%	80.62%	69.07%	60.35%	67.78%	28.17%	54.79%	-
PVNet (ResNet101)	48.81%	0.00%	42.15%	**74.22%**	69.40%	38.16%	80.23%	68.20%	61.80%	76.16%	37.63%	57.65%	-
PVNet (VGG16)${}_{IoU}$	30.53%	**4.00%**	24.81%	51.10%	48.57%	20.89%	66.31%	48.82%	**43.50%**	55.90%	19.37%	42.11%	-
PVNet (ResNet101)${}_{IoU}$	**36.85%**	0.00%	**26.77%**	**54.88%**	**54.77%**	**21.52%**	**66.43%**	**53.15%**	43.00%	**65.00%**	**23.90%**	**44.24%**	-

**Table 4:** Comparison of the class-wise accuracy on the NYU V2 dataset. Some of the methods in Table 2 do not provide the class-wise accuracy; hence, they are omitted here. The class-wise IoU of the Pixel-Voxel network (PVNet) are also provided. The methods with † take advantage of the data from multiple views. The best performance among the compared methods is marked as bold.

Category	Bed	Books	Ceiling	Chair	Floor	Furniture	Objects	Painting	Sofa	Table	TV	Wall	Window	Mean
Hermans et al. [21] †	68.4%	45.4%	**83.4%**	41.9%	91.5%	37.1%	8.6%	35.8%	28.5%	27.7%	38.4%	71.8%	46.1%	48.0%
SemanticFusion [22] †	62.0%	58.4%	43.3%	59.5%	92.7%	**64.4%**	58.3%	65.8%	48.7%	34.3%	34.3%	86.3%	62.3%	59.2%
PVNet (VGG16) †	**74.85%**	49.93%	82.18%	78.67%	**98.82%**	63.43%	52.57%	63.06%	**70.41%**	74.48%	73.48%	**94.85%**	74.98%	73.21%
PVNet (ResNet101) †	73.85%	**59.60%**	76.14%	**81.99%**	98.33%	58.82%	**59.19%**	**66.27%**	64.07%	**78.41%**	**79.67%**	94.53%	**76.66%**	**74.43%**
PVNet (VGG16)${}_{IoU}$ †	**64.17%**	33.34%	**64.05%**	64.25%	**90.39%**	**49.27%**	40.95%	45.17%	**54.78%**	62.83%	**52.31%**	80.62%	58.87%	58.54%
PVNet (ResNet101)${}_{IoU}$ †	63.09%	**38.35%**	61.16%	**68.58%**	89.66%	48.07%	**44.34%**	**50.39%**	50.89%	**63.48%**	49.97%	**81.51%**	**61.40%**	**59.30%**

**Table 5:** The average inference runtime of Pixel-Voxel Net (PVNet) using different sizes of data.

Network on the Different Sizes of Data	Inference Runtime	
	Full Size	Half Size
PVNet (VGG-16)	0.176s	0.075s
PVNet (ResNet101)	0.310s	0.111s

**Table 6:** The declining performance of Poxel-Voxel Net (PVNet) using half-sized data. △ represents the declining performance (in percentage) with half-sized data compared to that with full-sized data.

Network on the Half Size Data	SUN RGB-D			NYU V2		
	△ Pixel acc.	△ Mean acc.	△ Mean IoU	△ Pixel acc.	△ Mean acc.	△ Mean IoU
PVNet (VGG-16)	−1.35%	−1.87%	−1.59%	−1.08%	−0.62%	−1.53%
PVNet (ResNet101)	−1.16%	−2.34%	−1.94%	−1.41%	−0.84%	−1.96%
